# Postoperative Pharyngolaryngeal Adverse Events with Laryngeal Mask Airway (LMA Supreme) in Laparoscopic Surgical Procedures with Cuff Pressure Limiting 25 cmH_2_O: Prospective, Blind, and Randomised Study

**DOI:** 10.1155/2014/709801

**Published:** 2014-03-17

**Authors:** Joo-Eun Kang, Chung-Sik Oh, Jae Won Choi, Il Soon Son, Seong-Hyop Kim

**Affiliations:** ^1^Department of Anaesthesiology and Pain Medicine, Konkuk University Medical Center, Konkuk University School of Medicine, 120-1 Neungdong-ro (Hwayang-dong), Gwangjin-gu, Seoul 143-729, Republic of Korea; ^2^Institute of Biomedical Science and Technology, Konkuk University School of Medicine, Seoul, Republic of Korea

## Abstract

To reduce the incidence of postoperative pharyngolaryngeal adverse events, laryngeal mask airway (LMA) manufacturers recommend maximum cuff pressures not exceeding 60 cmH_2_O. We performed a prospective randomised study, comparing efficacy and adverse events among patients undergoing laparoscopic surgical procedures who were allocated randomly into low (limiting 25 cmH_2_O, L group) and high (at 60 cmH_2_O, H group) LMA cuff pressure groups with LMA Supreme. Postoperative pharyngolaryngeal adverse events were evaluated at discharge from postanaesthetic care unit (PACU) (postoperative day 1, POD 1) and 24 hours after discharge from PACU (postoperative day 2, POD 2). All patients were well tolerated with LMA without ventilation failure. Before pneumoperitoneum, cuff volume and pressure and oropharyngeal leak pressure (OLP) showed significant differences. Postoperative sore throat at POD 2 (3 versus 12 patients) and postoperative dysphagia at POD 1 and POD 2 (0 versus 4 patients at POD 1; 0 versus 4 patients at POD 2) were significantly lower in L group, compared with H group. In conclusion, LMA with cuff pressure limiting 25 cmH_2_O allowed both efficacy of airway management and lower incidence of postoperative adverse events in laparoscopic surgical procedures. This clinical trial is registered with KCT0000334.

## 1. Introduction

The cuff inflation of endotracheal tube (ETT) is essential to achieve a seal between ETT and tracheal wall. It makes no air leak at airway pressure required for positive pressure ventilation and lung protection from aspiration. The ETT cuff pressure is recommended as below 25–35 cmH_2_O to prevent the reduction of perfusion pressure in airway mucosa [1]. A higher cuff pressure in laryngeal mask airway (LMA) is permitted since the device is not located in trachea, surrounded by cartilages. The LMA Supreme (Intavent Orthofix, Maidenhead, UK) cuff pressure is recommended as below 60 cmH_2_O by the manufacturer [2]. However, Zhang et al. showed that LMA Supreme cuff pressure of 80 cmH_2_O was not associated with a greater incidence of postoperative pharyngolaryngeal adverse events, compared with 40 or 60 cmH_2_O [3]. In clinical practices, the cuff inflation of LMA is performed by single or multiple injections with 20–50 mL syringes, inflated with air, to achieve a tight seal and the LMA cuff pressure frequently exceeds 60 cmH_2_O [4]. However, several studies have reported that the reduction of cuff pressure of LMA resulted in fewer pharyngolaryngeal complications [5–7]. Brimacombe et al. also reported that small inflated cuff volume of LMA showed lower incidence of sore throat and dysphagia, comparing with high inflated cuff volume [8]. However, the studies have not been performed with LMA Supreme.

Therefore, we hypothesized that the LMA cuff pressure limiting 25 cmH_2_O could reduce the postoperative pharyngolaryngeal adverse events, compared with 60 cmH_2_O by the manufacturer, using LMA Supreme. The study was designed to compare the postoperative pharyngolaryngeal adverse events between the LMA cuff pressure limiting 25 cmH_2_O (L group) and at 60 cmH_2_O (H group). We also investigated the safety and efficacy of cuff pressure limiting 25 cmH_2_O in laparoscopic surgical procedures.

## 2. Materials and Methods

The study was approved by the Institutional Review Board (KUH1160036 granted by the Institutional Review Board of Konkuk University Medical Center, Seoul, Korea) and registered at KCT0000334. Written informed consents were obtained from the patients and the study was conducted in a prospective, blind, and randomised fashion.

### 2.1. Study Population

Patients undergoing laparoscopic surgical procedures were enrolled. Patients were excluded if the following criteria were present: (1) neurological or psychiatric disorders, (2) vocal cord paralysis, (3) recent history of respiratory infection (within 1 month), and (4) allergy to egg or soybean oil because of risk of allergy to propofol. The patients were randomly allocated before anaesthesia induction into either L group or H group by sealed envelope containing the group assignment.

### 2.2. Anaesthesia Technique

The anaesthesia technique was standardised. The patient arrived at the operating room without premedication. After establishing routine patient monitoring, anaesthesia was induced by an attending anaesthesiologist who was blind to the study. The following anaesthesia protocol was requested: lidocaine 0.5 mg/kg was administered to decrease pain from propofol injection, followed by intravenous propofol 2 mg/kg for anaesthesia induction and a target plasma concentration of remifentanil of 5 ng/mL, with target-controlled infusion device (Orchestra Base Primea, Fresenius Vial, Brézins, France), according to the Minto model [9]. The remifentanil of 5 ng/mL was maintained until the end of surgery. Rocuronium 0.6 mg/kg was administered for muscle relaxation after onset of deep sedation under the guidance of peripheral neuromuscular transmission (NMT) monitoring. The LMA insertion was performed at train-of-four count of 0. The cuff of the LMA was completely deflated prior to insertion and its dorsal surface was lubricated with jelly. LMA size was determined by patient body weight in accordance with the manufacturer guideline: <50 kg, size 3; 50–70 kg, size 4; 70–100 kg, size 5. The LMA cuff was inflated with air using a 50-mL syringe and the cuff pressure was adjusted with an aneroid manometer (Control Inflator Cuff Pressure manometer, VBM Medizintechnik GmbH, Germany) according to group allocation. The cuff pressure in L group was checked at the pressure to maintain the effective ventilation limiting 25 cmH_2_O. The inflated cuff volumes and pressures were recorded along with the number of attempts until successful LMA insertion. An attempt was defined by LMA placement in the mouth. The successful LMA insertion was defined as synchronised expansion of chest wall with unobstructed inspiratory/expiratory flow, normal capnographic tracing at positive pressure ventilation, and no-audible leak at LMA cuff just after LMA insertion. If these criteria were not met after 3 attempts at LMA insertion, the airway was secured according to the decision of the attending anaesthesiologists and the case was defined as an LMA insertion failure. The causes of LMA insertion failure were checked (insertion failure into airway, persistent air leak or ineffective ventilation). After LMA insertion, the patient's head was placed in a neutral position and the oropharyngeal leak pressure (OLP: the pressure at which a gas leak occurs around the airway device) was checked by closing the adjustable pressure-limiting valve of the anaesthesia circuit with manual ventilation at a fixed gas flow of 4 L/min. The OLP was determined when a sound was detected around the mouth by auscultation in equilibrium with the airway pressure of the anaesthesia circuit. If the no sound was heard above 40 cmH_2_O, the OLP was reported as 40 cmH_2_O. The airway pressure was not allowed to exceed 30 cmH_2_O. Anaesthesia was maintained with sevoflurane, titrated to maintain bispectral index values between 40 and 60. During anaesthesia, minimal and maximal end expiratory concentrations of sevoflurane were recorded with a gas analyser. After anaesthesia induction, volume-controlled ventilation with tidal volume 6 mL/kg of ideal body weight and no positive end-expiratory pressure was applied. The respiratory rate was adjusted to keep the partial pressure of end-tidal carbon dioxide between 35 and 40 mmHg. Additional rocuronium was administered under the guidance of the peripheral NMT monitoring. Pneumoperitoneum was generated by carbon dioxide insufflation to a maximum insufflation pressure of 12 mmHg. The LMA cuff pressure was checked with the aneroid manometer immediately after carbon dioxide insufflation and recorded. Peak and mean airway pressures were checked and recorded before carbon dioxide insufflation and rechecked and recorded after ventilator setting to maintain partial pressure of end-tidal carbon dioxide between 35 and 40 mmHg with adjustment of respiration rate but not tidal volume. Sevoflurane and remifentanil were discontinued at the end of the surgery and intravenous ketorolac 0.5 mg/kg was administered for postoperative pain control at operation site. Residual neuromuscular paralysis was antagonised with neostigmine 0.05 mg/kg and glycopyrrolate 0.01 mg/kg under the guidance of the peripheral NMT monitor. Pharyngeal suctioning was not routinely performed. The LMA cuff was completely deflated and the deflated cuff volume was checked using a 50-mL syringe. After LMA removal, the device was inspected for gross blood on the inside or outside surface and patient was transferred to the postanaesthesia care unit (PACU).

If the situation of ventilation failure occurred during the anaesthesia, the airway was secured according to the decision of the attending anaesthesiologists and the case was checked.

### 2.3. Postoperative Evaluation

Postoperative pain at operation site was assessed at the same points by using a visual analogue scale (VAS) that ranged from 0 to 100 mm with 0 = no pain and 100 = worst pain imaginable. Ketorolac 0.5 mg/kg given intravenously in the PACU or general ward with maximum of 3 doses was given as needed for postoperative analgesia and recorded.

Postoperative pharyngolaryngeal adverse events were assessed: sore throat was defined as constant pain or discomfort in the throat independent of swallowing; dysphonia was defined as difficult speaking or pain on speaking; dysphagia was defined as difficulty or pain provoked by swallowing [8]. The incidence of postoperative pharyngolaryngeal adverse events was recorded at discharge from the PACU (POD 1) and 24 hours after discharge from the PACU (POD 2). Any complications related to LMA insertion, such as recurrent laryngeal nerve palsy, hypoglossal nerve palsy, lingual nerve palsy, and arytenoid cartilage dislocation, were also recorded at POD 1 and POD 2.

Postoperative nausea and vomiting (PONV) was assessed at POD 1 and POD 2 using a 3-point ordinal scale (0 = none, 1 = nausea, 2 = retching, 3 = vomiting) [10]. The severity of PONV was evaluated using a modified Rhodes index at POD 2 [11].

All data were collected by trained observers who did not participate in patient care and were blind to the allocation.

### 2.4. Statistics

The primary outcome variable was the incidence of sore throat at POD 2. In a pilot study with 20 patients undergoing laparoscopic surgical procedures with the LMA cuff pressure at 60 cmH_2_O (H group), sore throat occurred in 8 patients. A minimum detected difference of 75% in the incidence of sore throat between the groups was considered to be of clinical significance. The sample size of 49 was calculated with a power of 0.9 and an *α* value of 0.05. The data were analysed using Statistical Package for the Social Sciences ver. 11.0 software. The values between two groups were analysed using an unpaired chi-square test as parametric test or Fisher exact test as nonparametric test for categorical variables and Mann-Whitney Rank Sum test as nonparametric test for continuous variables. The intragroup changes between POD 1 and POD 2 were compared with paired *t*-test as parametric test or Wilcoxon Signed Rank test as nonparametric test. All data were expressed as the number of patients or mean ± standard deviation. A value of *P* < 0.05 was considered statistically significant.

## 3. Results

**Figure 1:**
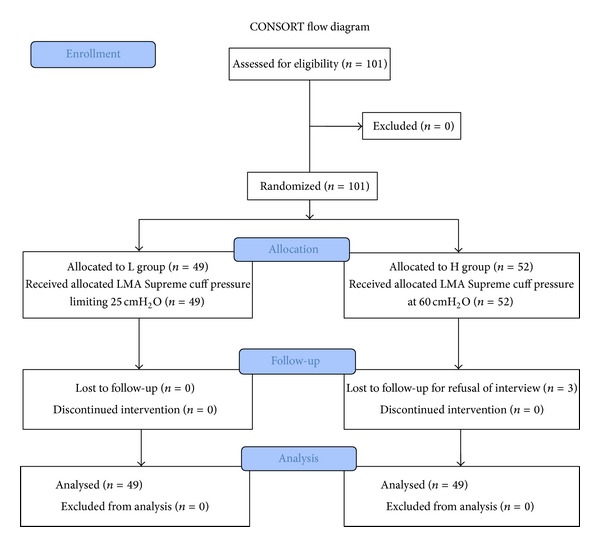
CONSORT flow diagram. LMA: laryngeal mask airway; L group: laryngeal mask airway (LMA) cuff pressure limiting 25 cmH_2_O group; H group: LMA cuff pressure at 60 cmH_2_O group.

From January to August 2012, a total of 101 patients consented to enrolment in the study. However, 3 patients in H group who refused interviews at POD 1 or POD 2 were excluded from the analysis. Therefore, a total of 98 patients were included in the analysis (Figure 1).

Demographic data between the groups were similar (Table 1).

**Figure 2:**
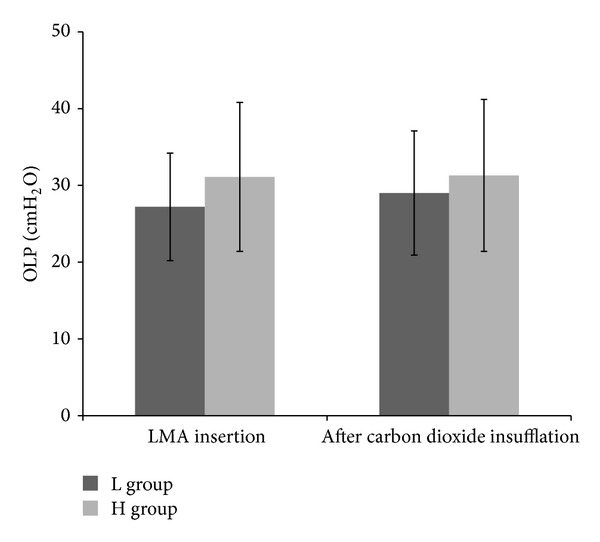
Changes of oropharyngeal leak pressure (OLP) before and after pneumoperitoneum with carbon dioxide (CO_2_) insufflation. LMA: laryngeal mask airway; L group: laryngeal mask airway (LMA) cuff pressure limiting 25 cmH_2_O group; H group: LMA cuff pressure at 60 cmH_2_O group.

Intraoperative measurements and recordings are presented in Table 2. The cuff volume and pressure before pneumoperitoneum showed significant difference between the two groups (volume: 19.5 ± 3.5 mL, L group versus 31.0 ± 3.6 mL, H group; *P* < 0.001; pressure: 18.6 ± 2.5 cmH_2_O, L group versus 55.9 ± 3.2 cmH_2_O, H group; *P* < 0.001). The OLP in the L group was significantly lower than that in the H group (27.2 ± 7.0 cmH_2_O versus 31.1 ± 9.7 cmH_2_O, *P* = 0.025) before pneumoperitoneum. There were no significant differences between the two groups in peak and mean airway pressures before pneumoperitoneum. After pneumoperitoneum with carbon dioxide insufflation, the LMA cuff pressure showed significant difference between the two groups (18.8 ± 4.0 cmH_2_O, L group versus 56.0 ± 4.8 cmH_2_O, H group; *P* < 0.001), but OLP and peak and mean airway pressures after pneumoperitoneum had no significant difference between two groups. Intragroup changes before and after pneumoperitoneum followed as below: changes of cuff pressure with carbon dioxide insufflation showed no differences in either group (*P* = 0.651 in L group and *P* = 0.937 in H group); OLP was significantly increased with carbon dioxide insufflation in the L group (27.2 ± 7.0 to 29.0 ± 8.1, *P* = 0.028) but had no significant change in the H group (31.1 ± 9.7 to 31.3 ± 9.9, *P* = 0.653) (Figure 2); peak and mean airway pressures were significantly increased after carbon dioxide insufflation in both groups (*P* < 0.0001). The cuff volume after LMA removal was significantly lower in the L group than in the H group (19.5 ± 3.5 mL, L group versus 31.4 ± 3.5 mL, H group; *P* < 0.0001) with no changes in cuff volumes at LMA insertion in either group.

All patients tolerated LMA well and there were no cases of ventilation failure. Three patients in each group had visible blood inside or outside the LMA device after LMA removal.

Details of postoperative pharyngolaryngeal adverse events are presented in Table 3. Postoperative sore throat at POD 2 was significantly lower in the L group (6.1%) than in the H group (24.5%) (*P* = 0.012). Postoperative dysphagia at POD 1 and POD 2 was also lower in the L group (0.0%) than in the H group (8.2%) (*P* = 0.041). One patient in the H group had dysphonia at POD 2, but there was no significant difference between groups. No complications related to LMA insertion were reported in either group.

The incidence of PONV was 18.4% in the L group and 24.5% in the H group at POD 1 and 14.3% (L group) and 24.5% (H group) at POD 2. However, the incidence of PONV assessed using a three-point ordinal scale and Rhodes index at POD 1 and POD 2 showed no significant differences between the two groups (Table 4).

## 4. Discussion

The present study demonstrated that LMA cuff pressure limiting 25 cmH_2_O decreased the incidence of postoperative pharyngolaryngeal adverse events, specifically sore throat and dysphagia, without intraoperative ventilatory failure with a lower OLP, using LMA Supreme.

As cuff pressure of ETT or LMA devices increases, perfusion of the airway mucosa is progressively decreased, which results in postoperative pharyngolaryngeal adverse events [12]. Therefore, limiting cuff pressures of airway devices can reduce postoperative pharyngolaryngeal adverse events [5–7]. Seet et al. performed a study similar to the present study and determined that LMA cuff pressures below 40 mmHg (60 cmH_2_O) were associated with reduced incidence of postoperative pharyngolaryngeal complications [4], but 60 cmH_2_O was still the higher limit in terms of airway perfusion pressure. Seet et al. had a lower incidence of sore throat (3.1% in 97 patients) and dysphagia (2.1% in 97 patients) at postoperative 24 hours in the pressure limiting group, compared with H group applying the same cuff pressure in the present study (sore throat of 24.5% and dysphagia of 8.2% in 49 patients). The cause would be associated with characteristics of postoperative analgesic. Seet et al. used opioid (fentanyl 25 *μ*g) for postoperative analgesic, while we used a nonsteroidal anti-inflammatory drug (NSAID, ketorolac 0.5 mg/kg). The low potency of the NSAID compared with the opioid [13] could have resulted in the higher incidences of sore throat and dysphagia in the present study. Zhang et al. showed that LMA Supreme cuff pressure of 80 cmH_2_O was not associated with higher incidence of postoperative pharyngolaryngeal adverse events [3]. Zhang et al. also used an opioid (fentanyl 25 *μ*g) as the postoperative analgesic. The higher incidence of sore throat at POD 2 than POD 1 would be associated with recovery from anaesthesia. The concern of postoperative pain at operation site would be converted into pain at other sites as the patient recovered from anaesthesia.

Regarding dysphonia, the LMA is a supraglottic airway device and the occurrence of dysphonia is rare. However, there is risk that a pressure neuropraxia from the LMA cuff can result in dysphonia [14–16]. The portion of the recurrent laryngeal nerve that is vulnerable to damage by the LMA is in the cricoid cartilage at the lower part of the piriform fossa [15]. When the LMA is precisely positioned, its tip will be resting against the upper oesophageal sphincter with the sides facing the piriform fossa [17]. Seet et al. reported dysphonia of 4.1% in pressure limiting group and 6.8% in routine care group. However, the LMA cuff pressure limiting 25 cmH_2_O in the present showed no occurrence of dysphonia. It would demonstrate that the cuff pressure of LMA could influence the occurrence of dysphonia.

The airway security between airway device and pharynx must be firstly considered before the postoperative pharyngolaryngeal adverse events. We demonstrated that the limited cuff pressure with 25 cmH_2_O safely secured airway, especially in laparoscopic surgical procedures. LMA use in laparoscopic surgical procedures has previously been restricted or contraindicated [18]. However, several researchers have proven the safety of LMA in laparoscopic surgical procedures [19, 20]. When an LMA Supreme is correctly positioned, the tip of the device serves as second seal at the top of the oesophagus over upper oesophageal sphincter [2, 21]. In the present study, a peak airway pressure not exceeding 20 cmH_2_O with an OLP of about 30 cmH_2_O and maintenance of effective ventilation after pneumoperitoneum in both groups was taken to indicate that the LMA was correctly positioned, although the visible evaluation for correct position of LMA was not performed. Gastric distension is a main cause in PONV [22]. The insignificant difference in the incidence of PONV between the two groups in the present study indicated that the limited cuff pressures with 25 cmH_2_O had an effective sealing effect of the airway, comparable with the higher cuff pressure at 60 cmH_2_O.

However, one consideration remains. OLP in both groups was increased after carbon dioxide insufflation. This was thought to be associated with the increased intra-abdominal pressure causing a morphologic change of the pharynx at the well-fitted LMA or a change of the LMA cuff at the well-fitted pharynx. Either way, the change of pharynx or LMA cuff might have resulted in the increased OLP. In the present study, we measured the cuff volume at LMA insertion and LMA removal. Almost the same volume at LMA insertion and LMA removal could rule out the effect of carbon dioxide diffusion into cuff of LMA with pneumoperitoneum.

Two limitations had to be considered. Firstly, the incidence of sore throat at POD 2 with 24.5% in H group was different with the pilot study (40% with 8 patients from 20 patients). The difference would need more or less sample size, although the sample size with 47 patients for each group in the present study was larger than the sample size with 40 patients for each group in Zhang at al. [3]. Lastly, the majority of the patients enrolled in the present study were undergoing laparoscopic cholecystectomy. For better visualisation, the standard position for this procedure is head-up and left-side tilted. This position would lessen the effects of the pneumoperitoneum on the LMA cuff. In addition, there were no extremely obese patients included in the present study. Cuff pressures in extremely obese patients in Trendelenburg position would be strongly influenced and would be expected to yield different results than those demonstrated here. Shorter anaesthesia time may also have influenced the results of the present study, although an LMA is usually not chosen for airway management when longer anaesthesia times are anticipated [18].

## 5. Conclusions

LMA cuff pressure limiting 25 cmH_2_O allowed safe airway management with secure airway and lower incidence of postoperative pharyngolaryngeal adverse events compared with cuff pressure at 60 cmH_2_O, using LMA Supreme.

## Figures and Tables

**Table 1:** Demographic data.

	L group	H group	*P*
Gender (M/F)	18/31	18/31	1.00
Age (yrs)	42 ± 15	41 ± 14	0.828
Height (cm)	164 ± 8	163 ± 7	0.468
Weight (kg)	64 ± 13	65 ± 11	0.592
Operation			0.686
Appendectomy	6	9	
Cholecystectomy	34	34	
Gynecological	9	6	
CO_2_ pr (mmHg)	12	12	
Time (min)			
Anesthesia	86 ± 31	86 ± 32	0.962
Operation	56 ± 30	58 ± 31	0.767
LMA size			0.299
Size 3	7	3	
Size 4	36	39	
Size 5	6	7	
Attempt number			0.399
1	47	45	
2	2	4	
3	0	0	
Insertion failure			—
Into airway	0	0	—
Air leak	0	0	—
Ineffective V	0	0	—
Blood on LMA	3	4	0.698
Sevoflurane			
Min (%)	0.9 ± 0.2	1.0 ± 0.4	0.76
Max (%)	1.7 ± 0.3	1.6 ± 0.3	0.63
Op site pain			
POD 1	40 ± 6	32 ± 5	0.86
POD 2	40 ± 8	34 ± 7	0.30
Ketorolac (mg)			
~POD 1	16 ± 17	12 ± 17	0.88
POD 1~POD 2	11 ± 16	7 ± 14	0.80

Values are expressed as number of patients or mean ± standard deviation.

L group: laryngeal mask airway (LMA) cuff pressure limiting 25 cmH_2_O group; H group: LMA cuff pressure at 60 cmH_2_O group; M: male; F: female; CO_2_ pr: insufflated carbon dioxide pressure for pneumoperitoneum, time, attempt number, and attempt number for successful LMA insertion; Into airway: failure into airway; Air leak: persistent air leak; Ineffective V: ineffective ventilation; Blood stained LMA: the presence of visible blood inside or outside of LMA; Min (%): minimal end expiratory concentrations of sevoflurane concentration (%); Max (%): maximal end expiratory concentration of sevoflurane (%); Op; operation; POD 1: postoperative day 1 (discharge from postanaesthetic care unit (PACU)); POD 2: postoperative day 2 (24 hours after discharge from PACU); ~POD 1: from end of surgery to discharge from PACU; POD 1~POD 2: from POD 1 to POD 2.

**Table 2:** Laryngeal mask airway (LMA) parameters compared between low and high LMA cuff pressures.

	LMA insertion		*P*	After CO_2_		*P*	LMA removal		*P*
	L group	H group		L group	H group		L group	H group	
LMA cuff									
Vol (ml)	19.5 ± 3.5	31.0 ± 3.6	<0.001	—	—	—	19.5 ± 3.5	31.4 ± 3.5	<0.001
Pr (cmH_2_O)	18.6 ± 2.5	55.9 ± 3.2	<0.001	18.8 ± 4.0	56.0 ± 4.8	<0.001	—	—	—
OLP (cmH_2_O)	27.2 ± 7.0	31.1 ± 9.7	0.025	29.0 ± 8.1	31.3 ± 9.9	0.208	—	—	—
Airway pr (cmH_2_O)									
Peak	12.1 ± 2.6	13.0 ± 2.6	0.106	17.0 ± 4.1	18.4 ± 3.8	0.106	—	—	—
Mean	5.3 ± 0.9	5.6 ± 1.2	0.109	6.5 ± 1.3	7.0 ± 1.5	0.154	—	—	—

Values are expressed as mean ± standard deviation.

After CO_2_: after carbon dioxide insufflation; L group: laryngeal mask airway (LMA) cuff pressure limiting 25 cmH_2_O group; H group: LMA cuff pressure at 60 cmH_2_O group; Vol: volume of air for LMA cuff inflation; Pr: pressure of air for LMA cuff inflation; OLP: oropharyngeal leak pressure; Airway pr: airway pressure.

**Table 3:** Postoperative pharyngolaryngeal adverse events.

	POD 1		*P*	POD 2		*P*
	L group	H group		L group	H group	
Sore throat	2	6	0.140	3	12	0.012
Dysphonia	0	0	—	0	1	0.315
Dysphagia	0	4	0.041	0	4	0.041
Cx	0	0	—	0	0	—

Values are expressed as number of patients.

L group: laryngeal mask airway (LMA) cuff pressure limiting 25 cmH_2_O group; H group: LMA cuff pressure at 60 cmH_2_O group; POD 1: postoperative day 1 (discharge from postanaesthetic care unit); POD 2: postoperative day 2 (24 hours after discharge from postanaesthetic care unit); Cx: any complications related with laryngeal mask airway insertion.

**Table 4:** Postoperative nausea and vomiting (PONV).

	POD 1		*P*	POD 2		*P*
	L group	H group		L group	H group	
PONV			0.461			0.509
None	40	37		42	37	
Nausea	7	9		3	6	
Retching	2	3		3	3	
Vomiting	0	0		1	3	
Rhodes index	0.6 ± 1.7	0.8 ± 1.6	0.592	1.3 ± 4.0	2.0 ± 4.8	0.443

Values are expressed as number of patients or mean ± standard deviation.

L group: laryngeal mask airway (LMA) cuff pressure limiting 25 cmH_2_O group; H group: LMA cuff pressure at 60 cmH_2_O group; POD 1: postoperative day 1 (discharge from postanaesthetic care unit); POD 2: postoperative day 2 (24 hours after discharge from postanaesthetic care unit).
